# Perception of COVID-19 Pandemic by Brazilian People With Parkinson’s Disease and Multiple Sclerosis

**DOI:** 10.3389/fpsyg.2022.718313

**Published:** 2022-05-19

**Authors:** Lucas Simieli, Felipe B. Santinelli, Elisa C. Costa, Marina H. Kuroda, Lorena R. Oliveira, Tiago Penedo, Julia Pilon, Aline P. B. Silveira, Iramaia S. A. Assis, Erica Tardelli, Erika Okamoto, Fabio A. Barbieri

**Affiliations:** ^1^Graduate Program in Movement Sciences, Human Movement Research Laboratory (MOVI-LAB), Department of Physical Education, School of Science, São Paulo State University (UNESP), Bauru, Brazil; ^2^REVAL Rehabilitation Research Center, Faculty of Rehabilitation Sciences, Hasselt University, Hasselt, Belgium; ^3^Associação Brazil Parkinson (ABP), São Paulo, Brazil

**Keywords:** lockdown 2020, COVID-19, neurodegenerative diseases, neuropsychological, Brazil

## Abstract

COVID-19 in Brazil is threatening, and it has forced the government to adopt partial lockdown as a strategy to stop the spread of the virus in the first wave of pandemic (March 2020). These preventive measures during the COVID-19 pandemic may affect the motor and non-motor symptoms in people with Parkinson’s disease (PD) and Multiple sclerosis (MS). Thus, the purpose of this study was to investigate the perception during the first wave of COVID-19 pandemic lockdown on motor and non-motor symptoms, and also measure physical activity level, quality of life, and sleep quality in Brazilian people with PD and MS. One hundred and fifty-three participants (PD-97 and MS-56) answered an online survey to identify the perception of motor and non-motor symptoms, and characterize the physical activity level, and quality of life and sleep in these neurological Brazillian population. During the beginning of pandemic lockdown in Brazil, our results indicated that 69% of people with PD and 55% of people with MS reported worse on motor aspects and lower amount of physical activity performed. Also, 75.2% (PD) and 92.9% (MS) of our cohort were considered inactive or sedentary. Based on the perception and behavior of the population studied, people with PD and MS should be encouraged to perform more physical activity in order to reduce the effects of isolation in motor and non-motor aspects of the diseases. Teleinterventions, such as home-based exercise, should be included in the new routine of people with PD and MS to reduce the impacts of lockdown and to maintain quality of life at a good level.

## Introduction

The number of cases and deaths due to the new coronavirus (COVID-19) in Brazil is threatening--more than twenty million and 560 thousand, respectively.^1^ To stop the spread of the virus during the pandemic, governments worldwide had being implementing some initiatives including vaccines and mitigating the population mobility with full/partial lockdowns. Specifically in Brazil, local and state governments were using partial lockdowns allowing only essential economic segments to be opened and restricting the movement of the population. These preventive and security measures ensured the non-collapse of the health system and to prevent thousands of people to be infected or dead. However, lockdown may affect motor and non-motor symptoms (i.e., worse symptoms) in the short and long term in people with movement disorders, such as Parkinson’s disease (PD) and multiple sclerosis (MS). Also, the stay in lockdown requires social distancing, leading to reduced access to healthcare and group therapies (Haji Akhoundi et al., 2020) and significant bearing on the morbidity among those with PD and MS (Brown et al., 2020; Plagg et al., 2020; Prasad et al., 2020b).

Staying at home for a long period reduces the amount of physical activity performed and reduces social interactions and support. Physical activity is being shown to be associated with both PD (Nimwegen et al., 2011), and MS (Motl et al., 2005) severity, as well as acting as a potent non-pharmacological treatment for both diseases (Dalgas and Stenager, 2012; Motl, 2020). However, due to restrictions caused by the pandemic lockdown, the population is staying inside their homes for a longer time than habitual, thus decreasing the amount of physical activity. For instance, in a recent Italian survey with 75 people with MS (Motolese et al., 2020), the authors observed an increase in depressive symptoms, such as worse sleep quality, and higher fatigue symptoms during the lockdown. Similar results were observed for people with PD: individuals with a reduced amount of physical activity reported worsening of motor symptoms during the pandemic lockdown (Schirinzi et al., 2020), while individuals with PD which are more active (Santinelli et al., 2021). Also, longer periods without going out of the house could increase psychological stress, anxiety, and depression symptoms, consequently decreasing the quality of life and sleep quality (Yalachkov et al., 2019; Prasad et al., 2020b).

These routine changes require a flexible behavior to new circumstances, which is a cognitive operation that can be impaired in people with movement disorders (Helmich and Bloem, 2020). Those individuals with movement disorders are frail individuals with specific cognitive, motor, and behavioral symptoms with inherent problems of adapting to changes and environmental stressors (Baschi et al., 2020). People with PD have symptoms of cognitive and motor inflexibility, as a result of nigrostriatal dopamine depletion that forms the pathophysiological substrate of PD (Robbins and Cools, 2014); while immunotherapy might favor mental distress in people with MS (Costabile et al., 2020). Nevertheless, although some recent researches were made in different countries (e.g., Italy and Israel) with people with PD and MS, the pandemic lockdown may affect differently the populations in different countries. The culture, education, and the way to face the COVID-19 pandemic of the population in each country influence the effects of lockdown (Allain-Dupré et al., 2020). Thus, understanding the effects of lockdown and the COVID-19 pandemic should consider these aspects to improve the measures adopting in each country considering different aspects related to weather, culture, etc.

Therefore, through an online survey, we investigated the perception of Brazilian people with PD and MS related to motor and non-motor symptoms, the amount of physical activity level performed, quality of life, and sleep quality during the COVID-19 pandemic lockdown in the first semester of 2020. We further investigated the correlations between the outcomes in order to identify ways to improve possible problems suffered from this population during lockdown periods. As observed by previous studies (Helmich and Bloem, 2020; Motolese et al., 2020; Schirinzi et al., 2020), we expected that during lockdown both people with PD and MS would present lower amount of physical activity and relate worse in motor and non-motor symptoms.

## Materials and Methods

### Participants

Using an electronic survey (Google Forms), we reached 204 individuals with PD or MS. The individuals were invited to participate in the study *via* social media (Facebook^®^, Instagram^®^), e-mail, and WhatsApp. Movement Disorder Centers across Brazil made available the contact of the individuals or invited the patients across their social media. A total of 153 participants (people of PD = 97 individuals, and people of MS = 56 individuals) were included in the study if they presented diagnosis of PD or MS confirmed by contact with the patient or movement disorder center; the participant was excluded if they respond provide inadequate questionnaire answers (e.g., no answered questions). The University’s local Ethical Committee approved this research and all individuals consented to participate in the study (#32134620.0.0000.5398). The participants answered the survey from May 21st to June 13th of 2020.

### Survey Composition

This is a cross-sectional exploratory and descriptive study. The participants filled seven sections of an online survey. The participants took around 10–15 min to respond to all the survey.

In the first part, they consented digitally to participate in the study. Secondly, participants responded to questions about social-demographic data (e.g., height, weight), clinical aspects, and general COVID-19 information (e.g., perception of the pandemic, diagnostic, and time in lockdown). After, a self-reported of motor and non-motor symptoms during lockdown were obtained (questions about their perception of home confinement on PD and MS motor and non-motor symptoms).

After, the level of physical activity was obtained through the short version of the International Physical Activity Questionnaire (IPAQ)–Expressed as Metabolic equivalent of task (MET) (Matsudo et al., 2012). The MET was calculated according to the participants’ answers. To identify the presence of anxiety and depression, the Hospital Anxiety and Depression (HAD) (Andrews et al., 2006) was performed. Punctuation higher than 8 (for anxiety), and 9 (for depression) indicate the presence of anxiety and depression symptoms. To evaluate sleep quality, the Mini Sleep Questionnaire (MSQ) (Natale et al., 2014) was obtained, with punctuation ranging from 10 to 24 for a good sleep quality and higher than 31 points for severe sleep difficulties. Finally, each group also responded to a specific questionnaire for quality of the life. People with PD responded to the Parkinson’s Disease Questionnaire-8 (PDQ-8) (Jenkinson et al., 1997), while people with MS responded to the Multiple Sclerosis Quality of Life-29 (MSQOL-29) (Rosato et al., 2019). PDQ is specific for people with PD and provides information about mobility, activities of daily living, emotional wellbeing, stigma, social support, cognition, communication, and bodily discomfort. On the other hand, MSQOL-29 provides information about people with MS on physical function, sexual function, cognitive function, bodily pain, and health distress.

### Data Analysis

Survey results and demographic information were summarized using descriptive statistics. The data about age, body mass, height, and disease duration are expressed as the average of absolute values, while schooling, COVID-19 diagnostics, time in partial lockdown, the seriousness of the pandemic, as well as the self-reported motor and non-motor symptoms during the pandemic lockdown, are expressed in percentage. The PDQ-8, MSQOL-29, IPAQ, HAD-Anxiety, HAD-Depression, and MSQ were reported in their respective classification. In addition, the SPSS (V.26) software was used to perform Spearman rank correlations among the variables used and the correlations were separately between the groups. The *p*-value was set at *p* < 0.05.

## Results

### General Overview

Table 1 presents the average of absolute values of demographic and general information, percentage of the impact of the lockdown on motor and non-motor symptoms, and implications of COVID-19 on quality of life, level of physical activity, anxiety and depression, and sleep quality in people with PD and MS. None of both groups presented positive diagnostics of COVID-19. A total 78.5% the individuals were in partial lockdown for more than 7 weeks and 68.5% of the individuals consider that the COVID-19 pandemic was a very serious issue.

**TABLE 1:** Demographic details, perceived impact on motor and non-motor symptoms, and implications of COVID-19 on quality of life, level of physical activity, anxiety and depression, and sleep quality in people with Parkinson’s disease (PD) and multiple sclerosis.

	Parkinson’s disease *N* = 97	Multiple sclerosis *N* = 56	Total *N* = 153
**DEMOGRAPHIC AND GENERAL INFORMATION**			
Gender	51 females/46 males	44 females/12 males	95 females/58 males
Age (years)	67 ± 11	41 ± 13	54 ± 12
Body mass (kg)	71 ± 16	74 ± 16	72.5 ± 16
Height (m)	1.62 ± 0.09	1.66 ± 0.12	1.65 ± 0.05
Disease duration (years)	9 ± 7	9 ± 8	8.5 ± 7.5
Have you been intake the disease medicine? (%)	YES: 93.0/NO: 1.0/More than before: 4.0/No intake medication for PD: 0	YES: 87.4/NO: 9.0/More than before: 0/No intake medication for MS: 3.6	YES: 91.7/NO: 4.5/More than before: 2 /No intake medication: 1.8
**Schooling (%)**			
*Elementary school*	17.5	0	9.3
*High school*	31.0	26.8	28.9
*Higher education*	41.2	53.6	46.9
*Master’s degree or PhD*	10.3	19.6	15.0
COVID-2019 positive diagnostic (%)	YES: 0/MAYBE: 2.1/NO: 97.9	YES: 0/MAYBE: 1.8/NO: 98.2	YES: 0/MAYBE: 2.0/NO: 98
**Time in partial lockdown (%)**			
*None*	4.1	7.1	6.7
*1 to 2 weeks*	0	0	0.0
*3 to 4 weeks*	3.1	5.4	3.8
*5 to 7 weeks*	6.2	16.1	11.2
*More than 7 weeks*	**86.6**	**71.4**	**78.5**
**Which is the seriousness of the COVID-19 pandemic? (%)**			
*Very serious*	**69.1**	**67.9**	**68.5**
*Serious*	24.7	25.0	24.4
*More or less serious*	4.1	5.4	4.8
*Less serious*	2.1	1.8	2.0
*Not serious*	0	0	0.5
**IMPACT ON MOTOR AND NON-MOTOR SYMPTOMS**			
**Motor symptoms (compared to before COVID-19 pandemic)**			
*Reported worsening general motor performance*	**YES: 69.0%**/NO: 31.0%	**YES: 55.0%**/NO: 45.0%	**YES: 61.5%**/NO: 38.5%
*Reported worsening rest tremor*	YES: 31.0%/NO: 69.0%	YES: 26.8%/NO: 73.2%	YES: 28.9%/NO: 71.1%
*Reported increased number of freezing episodes*	YES: 23.7%/NO: 76.3%	YES: 25.0%/NO: 75.0%	YES: 25.4%/NO: 74.6%
*Reported increased of unbalance*	YES: 34.0%/NO: 66.0%	YES: 32.1%/NO: 67.9%	YES: 33.1%/NO: 66.9%
*Reported worsening bradykinesia*	**YES: 59.8%**/NO: 40.2%	**YES: 57.1%**/NO: 42.9%	**YES: 58.5%**/NO: 41.5%
*Reported falling*	YES: 32.0%/NO: 68.0%	YES: 16.1%/NO: 83.9%	YES: 24.1%/NO: 75.9%
*Number of falls (n)*	1.1 ± 3.0 (total = 110)	0.4 ± 1.0 (total = 21)	0.75 ± 2 (total = 131)
**Non-motor symptoms (compared to before pandemic)**			
*Reported increased feeling of isolation (loneliness)*	**YES: 44.3%**/NO: 55.7%	**YES: 57.1**/NO: 42.9	**YES: 51.2**/NO: 48.8
*Reported decreasing of body sensation*	YES: 9.3%/NO: 90.7%	YES: 26.8/NO: 73.2	YES: 18.1/NO: 81.9
*Reported worsening memory failure (forgetfulness)*	YES: 25.8%/NO: 74.2%	YES: 34.0/NO: 66.0	YES: 29.9/NO: 70.1
*Reported increased tired (fatigue)*	YES: 39.2%/NO: 60.8%	YES: 42.9/NO: 57.1	YES: 41.1/NO: 58.9
*Reported changes in sleep quality*	Got better: 8.2%/NO: 49.5% /**Got worse: 42.3%**	Got better: 12.5%/NO: 32.1% /**Got worse: 55.4%**	Got better: 9.9%/NO: 41.3% /**Got worse: 48.9%**
**SPECIFIC QUESTIONARIES**			
			**Literature**
PDQ-8 (quality of life)	32.1 ± 17.7 pts (from 0 to 84) Good: 53.6%/**Bad: 45.3%**	–	*Classification* >33.14 pts = Significant symptoms for bad quality of life
MSQOL-29 (quality of life)	–	59.3 ± 19.26% (from 19.9 to 94.8) **health physical (**54% Good**/46% Bad)** 52.69 ± 15.4% (from 12.47 to 78.79) **mental health (**31% Good**/69% Bad)**	*Classification* 0%–Bad 100%–Good >60%–considered Good
IPAQ (level of physical activity)	544.3 ± 711.3 MET-min (from 0 to 4246.2) Much active: 2.1%/Active: 22.7%/**Inactive: 68.1%/Sedentary: 7.1%**	204.9 ± 298.1 MET-min (from 0 to 1729.8) Much active: 0%/Active: 7.1%/**Inactive: 69.7%/Sedentary: 23.2%**	*Classification* >3000 pts = Much active; 600–3000 pts = Active; 1–599 pts = Inactive; 0 pts = Sedentary
HAD-A (anxiety)	7.6 ± 3.7 pts (from 1 to 19) **With symptoms: 45.3%**/No symptoms: 54.7%	9.0 ± 3.6 pts (from 0 to 18) **With symptoms: 67.8%**/No symptoms: 32.3%	*Classification* >8 pts = Significant symptoms
HAD-D (depression)	6.4 ± 4.1 pts (from 0 to 17) With symptoms: 29.9%/No symptoms: 70.1%	6.8 ± 4.2 pts (from 1 to 17) With symptoms: 32.1%/No symptoms: 67.9%	*Classification* >9 pts = Significant symptoms
MSQ (Mini Sleep Questionnaire)	36.5 ± 9.7 pts (from 17 to 57) Good sleep quality: 14.4% Possible mild sleep difficulties: 4.1% Moderate sleep difficulties: 7.2% **Severe sleep difficulties: 74.2%**	34.5 ± 10.1 pts (from 14 to 57) Good sleep quality: 16.1% Possible mild sleep difficulties: 14.3% Moderate sleep difficulties: 8.9% **Severe sleep difficulties: 60.7%**	*Classification* 10–24 pts = good sleep quality; 25–27 pts = possible mild sleep difficulties; 28–30 pts = moderate sleep difficulties; >31 pts = severe sleep difficulties

*Bold numbers indicate important findings.*

People with PD (69%) and MS (55%) reported worsening in general motor performance. The bradykinesia was the motor symptom reported with the biggest worsening by the individuals during partial lockdown (people with PD: 59.8%; people with MS: 57.1%). Also, higher loneliness was reported by both groups (people with PD: 42.3%, and people with MS: 55.4%).

Specific questionnaires showed that the quality of life was bad for people with PD (45.3%) and MS (physical health: 34%; mental health: 100%). Around 75.2 and 92.9% of the people with PD and MS, respectively, presented inactive or sedentary behavior. Anxiety was more predominantly in people with MS (67.8%) than in people with PD (45.3%). Finally, the sleep quality reduced during the partial lockdown, with 74.2% people with PD and 60.7% of people with MS reporting severe sleeping difficulties.

### Correlation Among Variables

**FIGURE 1:**
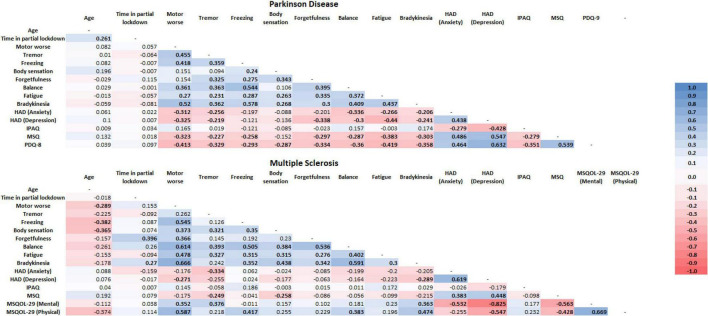
Spearman rank correlation among the variables used and separately between the groups. In bold is presented the significance difference. HAD, hospital anxiety and depression; IPAQ, international physical activity questionnaire; MSQ, mini sleep questionnaire; PDQ-8, Parkinson’s disease questionnaire-8; MSQOL-29, multiple sclerosis quality of life-29.

The Spearman’s rank correlation coefficient is presented in Figure 1 and the detailed statistical analysis (*p*-value) is presented as Supplementary Material.

### Parkinson’s Disease

The time in partial lockdown was not correlated with any variable measured in the present study. Oh the other hand, worse motor performance perceived by the people with PD was related with higher perceived in tremor, balance, bradykinesia, balance (*p* < 0.001) and fatigue (*p* < 0.008) worse, as well as with higher anxiety (*p* < 0.002) and depression (*p* < 0.001) symptoms, and lower sleep quality and quality of life (*p* < 0.001). Also, both lower sleep quality and quality of life was related with higher perceived worse in tremor (*p* < 0.026 and *p* < 0.001, respectively), freezing (*p* < 0.014 and *p* < 0.005, respectively), forgetfulness (*p* < 0.004 and *p* < 0.001, respectively), balance (*p* < 0.05 and *p* < 0.001, respectively), fatigue (*p* < 0.001), bradykinesia (*p* < 0.03 and *p* < 0.001, respectively), and anxiety and depression (*p* < 0.001) symptoms. For the last, those with PD who performed higher amounts of physical activity presented lower anxiety (*p* < 0.006) and depression (*p* < 0.001) symptoms, as well as better sleep quality (*p* < 0.006) and quality of life (*p* < 0.001).

### Multiple Sclerosis

Differently from those with PD and surprisely, people with MS which are in lockdown for shorter time related worse in forgetfulness (*p* < 0.003) and bradykinesia (*p* < 0.046). Moreover, people with MS that related worse in motor performance, also related worse in freezing, balance, bradykinesia, fatigue (*p* < 0.001), body sensation (*p* < 0.006) and forgetfulness (*p* < 0.007). In addition, worse perceived motor performance was related with depression symptom (*p* < 0.045) and with poor mental (*p* < 0.008) and physical (*p* < 0.001) quality of life. Similarly to the people with PD, poor sleep quality and quality of life was related with higher anxiety (MSQ:*p* < 0.004; MSQOL-29 mental: *p* < 0.001) and depression (MSQ, MSQOL-29 mental and physical: *p* < 0.001) symptoms.

## Discussion

The purpose of this study was to investigate the effects of the partial lockdown on the perception of motor and non-motor symptoms, the amount of physical activity level performed, quality of life, and sleep quality in Brazilian people with PD and MS. Most of the individuals (61.5% overall) perceived that the motor performance worse during the lockdown. This motor performance worse was related with a number of motor symptoms (e.g., tremor and bradykinesia) for both groups, with poor sleep quality and higher anxiety symptom (for people with PD) and also with higher depression symptom and poor quality of life (for both groups). Thus, our results suggest that despite the preventive and security measures being necessary to avoid the collapse of the health system and to prevent infections and deaths, people with PD and MS require further assistance (e.g., remote care) to reduce the effects observed during the pandemic lockdown. This is an opportunity to improve self-management strategies (e.g., remote exercise and cognitive therapy) that can help patients to better deal with the challenges of social distancing and other consequences of the COVID-19 pandemic.

During the partial lockdown, due to the COVID-19 pandemic, both people with PD and MS perceived a worse in motor performance. Our findings corroborated with previous studies that also indicated worsening in motor and non-motor symptoms in people with PD and MS (Baschi et al., 2020; Brown et al., 2020; Cilia et al., 2020). Regarding motor symptoms, people with PD and MS reported worsening mainly in motor performance and bradykinesia. The worsening of motor symptoms may be assigned to either the difficulty in accessing health care for a review of the disease’s severity or the inability to procure medication refills (Prasad et al., 2020a). In addition, the obligatory increase in sedentary lifestyle due to the COVID-19 confinement leads to a greater deterioration in motor symptoms (Prasad et al., 2020b; Schirinzi et al., 2020). Those motor symptoms are enough to create a fragile framework for these patients, increasing the risk of falling and the dependence on the caregiver to avoid execution fails. Specifically, the bradykinesia requires that the individuals start their diary routine activities (e.g., cooking, changing clothes, brush teeth) earlier than normal to have enough time to perform, thus changing routine which may be a problem for people with movement disorders (Helmich and Bloem, 2020). This change in the behavior increases the anxiety and stress of the individuals (Stojanov et al., 2020).

Loneliness, anxiety, and sleep quality were the main non-motor symptoms that worsened during the lockdown in Brazilian people with PD and MS. Again, it is well-known that physical inactivity is a risk factor for non-motor symptoms, including insomnia, anxiety, and constipation (Nimwegen et al., 2011). A previous study reported that the obligatory increase in sedentary lifestyle due to the COVID-19 lockdown may lead to greater deterioration in cognitive and behavioral functioning (Baschi et al., 2020). Also, worsening of non-motor symptoms may be attributable to worst acceptance, resilience, and understanding of the pandemic situation in individuals with neurological disease (Stojanov et al., 2020). For example, 70% of the people with MS reported a perceived risk of COVID-19 infection higher than the general population (Capuano et al., 2020). Besides, an increase in anxiety has been described concerning disease management and access to healthcare services during the COVID-19 pandemic (Stojanov et al., 2020). These factors seem to be related to low sleep quality during the lockdown (Kumar et al., 2020). Several factors have been proposed for sleep disturbances during home confinement that includes, but not limited to, the reduction of face-to-face social interactions, loneliness, emotional stress, and an increase in screen time (Altena et al., 2020; Voitsidis et al., 2020).

Motor and non-motor worsening presented above could explain the reduced quality of life reported by people with PD and MS. Lockdown forces people to stay at home, and in most cases, working in a completely different setting, thus increasing, among other things, the stress level, anxiety, and depression. Those non-motor symptoms impact directly the quality of life. However, a possible strategy to deal with these disturbances is to adopt new hobbies and meaningful activities during the COVID-19 lockdown.

The reduced level of physical activity–more than 75% of individuals were inactive or sedentary during lockdown–may explain negative changes in motor symptoms, non-motor symptoms, and quality of life in people with PD and MS during the lockdown. Physical exercise contributes to hormonal releases related to happiness, good sleep, movement, and neural protection (Reuter et al., 2011; Altena et al., 2020; Brown et al., 2020), which may facilitate adaptation during long periods that people must stay at home. Despite physical activity, our analysis also revealed a negative correlation between age and motor worsening, freezing and body sensation, indicating a greater impairment of isolation for older people with MS, related, probably, to aging process. In this regard, web-based exercise initiatives could be a successful strategy, to promote both health and fight against isolation issues. Those interventions, such as synchronous or asynchronous exercise programs, for people with PD and MS are important for making access to these interventions more accessible, particularly for those living in populated areas of the world as Brazil.

Our study has some limitations. First, survey completion was naturally limited to people who were healthy enough to log-in online and fill out a survey. In addition, participation in the survey might have been biased by the relationship between individual patients and the Movement Disorder Centers across Brazil, in which patients were more closely monitored than others because of the ongoing treatment being more likely to participate. Second, we were not able to evaluate the participants before the pandemic lockdown, which limits our results based only on an observational mode of the self-reported responses from the participants. Also, due to the cross-sectional design of the study, it is unclear whether the observed clinical worsening represents a transient or a persistent phenomenon. Finally, the current findings should be interpreted in the context of possible variability in the pandemic severity (the level of severity of the pandemic was different across Brazilian states), degree of lockdown (Brazil had partial lockdown with a moderate adhesion), patients’ perception, and cultural characteristics.

Our findings indicated a concern for the reported motor and non-motor symptoms by the individual, as well as the quality of life, and sleep quality in Brazilian people with PD and MS during the COVID-19 pandemic. Teleinterventions should be included in the new routine of people with PD and MS to reduce the impacts of the lockdown. Home-based exercises using bodyweight such as squats or using daily items (e.g., water bottles, school bags, books, etc.), dancing, and walking inside the house are good options to keep active and improving or maintaining the quality of life in a good level. Due to the long period of the COVID-19 pandemic in the world, and especially in Brazil, further studies should focus on the great impact on the long-term quality of life in people with movement disorders.

## Data Availability Statement

The raw data supporting the conclusions of this article will be made available by the authors, without undue reservation.

## Ethics Statement

The studies involving human participants were reviewed and approved by the UNESP/Bauru Local Ethics Committee–(#32134620.0.0000.5398). The patients/participants provided their written informed consent to participate in this study.

## Author Contributions

LS and FB: conceptualization, data curation, methodology, writing, and reviewing and editing. FS: data curation, writing, and reviewing and editing. EC, MK, LO, TP, JP, AS, and IA: writing and data curation. ET and EO: visualization and data curation. All authors listed have made a substantial, direct, and intellectual contribution to the work, and approved it for publication.

## Conflict of Interest

The authors declare that the research was conducted in the absence of any commercial or financial relationships that could be construed as a potential conflict of interest.

## Publisher’s Note

All claims expressed in this article are solely those of the authors and do not necessarily represent those of their affiliated organizations, or those of the publisher, the editors and the reviewers. Any product that may be evaluated in this article, or claim that may be made by its manufacturer, is not guaranteed or endorsed by the publisher.

## Footnotes

1
https://www.worldometers.info/coronavirus/country/brazil/
