# Supplementary material for: Perception of COVID-19 Pandemic by Brazilian People With Parkinson’s Disease and Multiple Sclerosis
Source: Front Psychol. 2022 May 19;13:718313. doi: 10.3389/fpsyg.2022.718313 (PMC9162171; doi:10.3389/fpsyg.2022.718313)
Supplement: Supplementary file 1 [file Data_Sheet_1.docx]

Presentation of p values for the correlation analysis for both Parkinson disease and Multiple Sclerosis patients.

**Parkinson Disease**

|  | **Age** | **Time in partial lockdown** | **Motor worse** | **Tremor** | **Freezing** | **Body sensation** | **Forgetfulness** | **Balance** | **Fatigue** | **Bradykinesia** | **HAD (Anxiety)** | **HAD (Depression)** | **IPAQ** | **MSQ** | **PDQ-9** |
| --- | --- | --- | --- | --- | --- | --- | --- | --- | --- | --- | --- | --- | --- | --- | --- |
| **Age** | - |  |  |  |  |  |  |  |  |  |  |  |  |  |  |
| **Time in partial lockdown** | **0.011** | - |  |  |  |  |  |  |  |  |  |  |  |  |  |
| **Motor worse** | 0.424 | 0.586 | - |  |  |  |  |  |  |  |  |  |  |  |  |
| **Tremor** | 0.925 | 0.541 | **<.001** | - |  |  |  |  |  |  |  |  |  |  |  |
| **Freezing** | 0.438 | 0.95 | **<.001** | **<.001** | - |  |  |  |  |  |  |  |  |  |  |
| **Body sensation** | 0.058 | 0.945 | 0.145 | 0.372 | **0.024** | - |  |  |  |  |  |  |  |  |  |
| **Forgetfulness** | 0.779 | 0.276 | 0.139 | **0.001** | **0.01** | **<.001** | - |  |  |  |  |  |  |  |  |
| **Balance** | 0.779 | 0.994 | **<.001** | **<.001** | **<.001** | 0.313 | **<.001** | - |  |  |  |  |  |  |  |
| **Fatigue** | 0.902 | 0.588 | **0.008** | **0.024** | **0.006** | **0.011** | **0.001** | **<.001** | - |  |  |  |  |  |  |
| **Bradykinesia** | 0.568 | 0.432 | **<.001** | **<.001** | **<.001** | **0.009** | **0.003** | **<.001** | **<.001** | - |  |  |  |  |  |
| **HAD (Anxiety)** | 0.554 | 0.835 | **0.002** | **0.012** | 0.062 | 0.397 | 0.052 | **<.001** | **0.009** | **0.043** | - |  |  |  |  |
| **HAD (Depression)** | 0.33 | 0.948 | **0.001** | **0.032** | 0.252 | 0.192 | **<.001** | **0.003** | **<.001** | **0.018** | **<.001** | - |  |  |  |
| **IPAQ** | 0.929 | 0.74 | 0.106 | 0.857 | 0.254 | 0.417 | 0.825 | 0.13 | 0.976 | 0.088 | **0.006** | **<.001** | - |  |  |
| **MSQ** | 0.197 | 0.86 | **0.001** | **0.026** | **0.014** | 0.144 | **0.004** | **0.005** | **<.001** | **0.003** | **<.001** | **<.001** | **0.006** | - |  |
| **PDQ-8** | 0.706 | 0.352 | **<.001** | **0.001** | **0.005** | **0.005** | **<.001** | **<.001** | **<.001** | **<.001** | **<.001** | **<.001** | **<.001** | **<.001** | - |

**Multiple Sclerosis**

|  | **Age** | **Time in partial lockdown** | **Motor worse** | **Tremor** | **Freezing** | **Body sensation** | **Forgetfulness** | **Balance** | **Fatigue** | **Bradykinesia** | **HAD (Anxiety)** | **HAD (Depression)** | **IPAQ** | **MSQ** | **MSQOL-29 (Mental)** | **MSQOL-29 (Physical)** |
| --- | --- | --- | --- | --- | --- | --- | --- | --- | --- | --- | --- | --- | --- | --- | --- | --- |
| **Age** | - |  |  |  |  |  |  |  |  |  |  |  |  |  |  |  |
| **Time in partial lockdown** | 0.898 | - |  |  |  |  |  |  |  |  |  |  |  |  |  |  |
| **Motor worse** | **0.032** | 0.264 | - |  |  |  |  |  |  |  |  |  |  |  |  |  |
| **Tremor** | 0.099 | 0.504 | 0.054 | - |  |  |  |  |  |  |  |  |  |  |  |  |
| **Freezing** | **0.005** | 0.537 | **<.001** | 0.367 | - |  |  |  |  |  |  |  |  |  |  |  |
| **Body sensation** | **0.007** | 0.598 | **0.006** | **0.019** | **0.011** | - |  |  |  |  |  |  |  |  |  |  |
| **Forgetfulness** | 0.255 | **0.003** | **0.007** | 0.296 | 0.172 | 0.097 | - |  |  |  |  |  |  |  |  |  |
| **Balance** | 0.054 | 0.056 | **<.001** | **0.003** | **<.001** | **0.005** | **<.001** | - |  |  |  |  |  |  |  |  |
| **Fatigue** | 0.266 | 0.495 | **<.001** | **0.015** | **0.022** | **0.022** | **0.043** | **0.002** | - |  |  |  |  |  |  |  |
| **Bradykinesia** | 0.194 | 0.046 | **<.001** | 0.076 | **0.01** | **0.001** | **0.011** | **<.001** | 0.026 | - |  |  |  |  |  |  |
| **HAD (Anxiety)** | 0.521 | 0.247 | 0.198 | **0.013** | 0.66 | 0.865 | 0.542 | 0.146 | 0.144 | 0.133 | - |  |  |  |  |  |
| **HAD (Depression)** | 0.582 | 0.901 | 0.045 | 0.061 | 0.865 | 0.205 | 0.651 | 0.231 | 0.102 | **0.032** | **<.001** | - |  |  |  |  |
| **IPAQ** | 0.77 | 0.959 | 0.29 | 0.673 | 0.182 | 0.984 | 0.912 | 0.936 | 0.209 | 0.832 | 0.85 | 0.192 | - |  |  |  |
| **MSQ** | 0.16 | 0.565 | **0.202** | 0.067 | 0.773 | 0.062 | 0.537 | 0.683 | 0.47 | 0.115 | 0.004 | **<.001** | 0.477 | - |  |  |
| **MSQOL-29 (Mental)** | 0.416 | 0.785 | **0.008** | 0.005 | 0.937 | 0.263 | 0.462 | 0.187 | 0.091 | **0.006** | **<.001** | **<.001** | 0.197 | **<.001** | - |  |
| **MSQOL-29 (Physical)** | **0.005** | 0.405 | **<.001** | 0.109 | **0.002** | 0.066 | 0.096 | **0.004** | 0.151 | **<.001** | 0.06 | **<.001** | 0.088 | **0.001** | **<.001** | - |
